# The Systemic Inflammatory Response to *Clostridium difficile* Infection

**DOI:** 10.1371/journal.pone.0092578

**Published:** 2014-03-18

**Authors:** Krishna Rao, John R. Erb-Downward, Seth T. Walk, Dejan Micic, Nicole Falkowski, Kavitha Santhosh, Jill A. Mogle, Cathrin Ring, Vincent B. Young, Gary B. Huffnagle, David M. Aronoff

**Affiliations:** 1 Division of Infectious Diseases, the University of Michigan School of Medicine, Ann Arbor, Michigan, United States of America; 2 Division of Pulmonary and Critical Care Medicine, the University of Michigan School of Medicine, Ann Arbor, Michigan, United States of America; 3 Department of Internal Medicine, the University of Michigan School of Medicine, Ann Arbor, Michigan, United States of America; 4 Department of Microbiology and Immunology, the University of Michigan School of Medicine, Ann Arbor, Michigan, United States of America; 5 Division of Infectious Diseases, Vanderbilt University Medical Center, Nashville, Tennessee, United States of America; 6 Department of Medicine, Vanderbilt University Medical Center, Nashville, Tennessee, United States of America; 7 Department of Microbiology and Immunology, Montana State University, Bozeman, Montana, United States of America; 8 Division of Infectious Diseases, Veterans Affairs Ann Arbor Healthcare System, Ann Arbor, Michigan, United States of America; Institute Pasteur, France

## Abstract

**Background:**

The systemic inflammatory response to *Clostridium difficile* infection (CDI) is incompletely defined, particularly for patients with severe disease.

**Methods:**

Analysis of 315 blood samples from 78 inpatients with CDI (cases), 100 inpatients with diarrhea without CDI (inpatient controls), and 137 asymptomatic outpatient controls without CDI was performed. Serum or plasma was obtained from subjects at the time of CDI testing or shortly thereafter. Severe cases had intensive care unit admission, colectomy, or death due to CDI within 30 days after diagnosis. Thirty different circulating inflammatory mediators were quantified using an antibody-linked bead array. Principal component analysis (PCA), multivariate analysis of variance (MANOVA), and logistic regression were used for analysis.

**Results:**

Based on MANOVA, cases had a significantly different inflammatory profile from outpatient controls but not from inpatient controls. In logistic regression, only chemokine (C-C motif) ligand 5 (CCL5) levels were associated with cases vs. inpatient controls. Several mediators were associated with cases vs. outpatient controls, especially hepatocyte growth factor, CCL5, and epithelial growth factor (inversely associated). Eight cases were severe and associated with elevations in IL-8, IL-6, and eotaxin.

**Conclusions:**

A broad systemic inflammatory response occurs during CDI and severe cases appear to differ from non-severe infections.

## Introduction

Antibiotic-associated diarrhea caused by the toxigenic, Gram-positive anaerobic bacterium *Clostridium difficile* has emerged over the past decade as a major nosocomial infection. It causes significant morbidity and mortality [1] and has been estimated to impose an excess cost of $4.8 billion per year in US acute-care facilities [2]. The clinical spectrum of *C. difficile* infection (CDI) is wide, ranging from asymptomatic colonization to mild diarrhea to fulminant colitis, sepsis, and death [3], [4]. In addition, a significant fraction of patients with CDI experience recurrent disease [5], [6]. The need for better preventive and therapeutic strategies against CDI has driven new studies into host-microbial interactions and disease pathogenesis.

The local immune response to CDI is characterized by neutrophil recruitment and acute inflammation [7], and new mouse models are facilitating detailed studies to model the onset, progression, and resolution of inflammatory responses during infection [8], [9], [10], [11], [12], [13]. There are several studies evaluating the presence of cytokines in fecal samples from affected patients [14], [15], however, few studies have explored systemic inflammatory responses to infection in humans. Defining characteristic changes in inflammatory mediators in the circulation of infected patients could reveal biomarkers (or sets of biomarkers) that provide prognostic and/or diagnostic information. Such information could also be used to predict the likelihood of therapeutic success or recurrence following treatment.

To address gaps in our understanding of systemic inflammatory responses to CDI, we measured a panel of inflammatory protein mediators (cytokines, chemokines, and growth factors) in the circulation of hospitalized CDI patients (cases), hospitalized patients with diarrhea who tested negative for CDI (inpatient controls), or asymptomatic outpatients (outpatient controls). In addition, we sought to compare systemic inflammatory responses in cases with severe CDI versus non-severe infection.

## Materials and Methods

### Ethics statement

This study was approved by the University of Michigan Institutional Review Board and written informed consent was obtained from all participants.

### Human subjects

**Figure 1:**
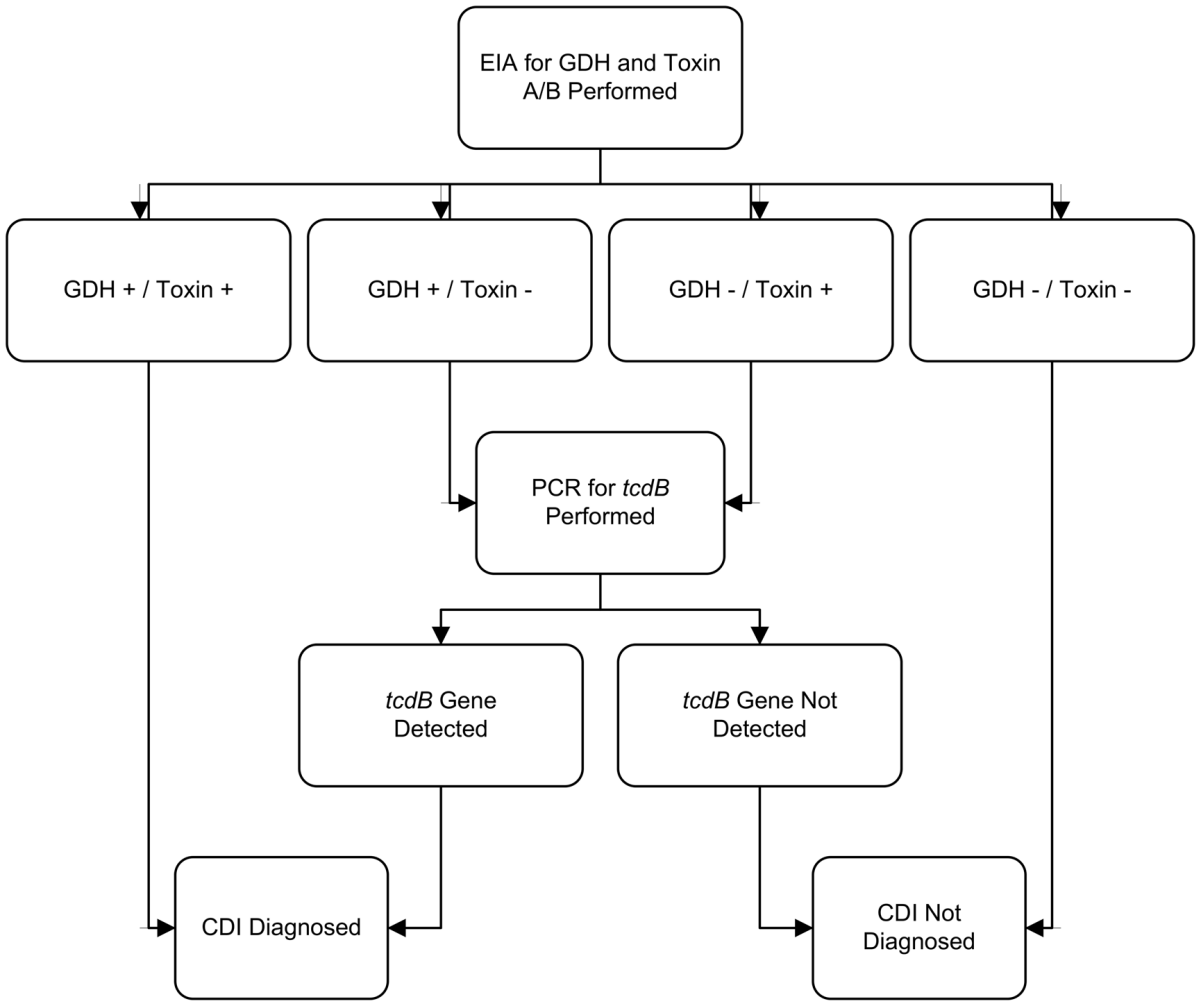
Testing algorithm for *Clostridium difficile* infection. This flow diagram illustrates this University of Michigan diagnostic testing algorithm for detecting toxigenic *Clostridium difficile* in stool. *Abbreviations*: CDI, *Clostridium difficile* infection; EIA, enzyme immunoassay; GDH, glutamate dehydrogenase; PCR, polymerase chain reaction.

The University of Michigan Health System (UMHS) has a 930-bed, tertiary care inpatient facility. The institution utilizes an electronic medical record (EMR) system providing access to patient records. Demographic information was extracted from the EMR and/or our study’s REDCap database [16], hosted at UMHS. Initial stool testing of inpatients was performed at the discretion of the inpatient care team. Inpatients stool samples sent for *C. difficile* testing were obtained from the microbiology laboratory sequentially. Testing was performed on stools using the C. DIFF QUIK CHEK COMPLETE test for *C. difficile* glutamate dehydrogenase (GDH) and toxins A or B (Techlab, Inc., Blacksburg, VA). All GDH^+^/toxin^−^ stool tests were subjected to analysis for the *tcdB* gene by real-time PCR (BD GeneOhm Cdiff Assay; Franklin Lakes, NJ) run on a Cepheid SmartCycler System (Cepheid, Sunnyvale, CA). An outline of our testing algorithm is shown in Figure 1. Attempts to confirm positive or negative *C. difficile* tests were performed using anaerobic culture on taurocholate-cycloserine-cefoxitin-fructose agar at 37°C followed by PCR to confirm taxonomy and presence of *C. difficile* toxin genes as previously described [17], [18], [19]. All patients were ≥ age 18 and not pregnant. Cases were hospitalized at UMHS, had diarrhea, and were identified by a positive test for *C. difficile* performed by the Clinical Microbiology Laboratory using the testing algorithm outlined in Figure 1. Inpatient controls were hospitalized patients with diarrhea that were suspected to have CDI by the primary team, but tested negative. Outpatient controls were non-hospitalized adults without diarrhea for at least the prior seven days recruited for study enrollment. Severe CDI was defined according to McDonald et al., as patients requiring intensive care unit admission due to CDI, undergoing interventional surgery to treat CDI, or death due to CDI within 30 days of diagnosis [20]. Other data regarding vital signs, laboratory measurements, proton pump inhibitor (PPI) use, and Charlson-Deyo scores [21], were extracted from the medical record by structured query and included if recorded within 48 hours of stool sample testing; these data were largely unavailable from the outpatient control group.

### Serum/plasma sampling

For cases and inpatient controls, serum or plasma was obtained within a median time of less than 24 hours of the lab result for presence of toxigenic *C. difficile*. Serum/plasma was obtained from outpatient controls at the time of enrollment. All samples were stored at −80°C until used for this study.

### Bead-based, multiplex antibody array for inflammatory mediators

An Invitrogen Multiplex Bead Immunoassay Kit (Human Cytokine 30-Plex Panel; Life Technologies, Grand Island, NY) was used to test serum/plasma samples according to the manufacturer’s instructions. A Luminex 200 dual laser detection system was used to analyze samples/standards. A list of the inflammatory mediators (cytokines, chemokines, and growth factors), and their standard abbreviations [22], are provided in Table 1.

**Table 1:** Thirty inflammatory mediators (cytokines, chemokines, and growth factors) measured in the circulation of study subjects.

Inflammatory Mediator	Alternate Name(s)/Abbreviation(s)
Vascular Endothelial Growth Factor (VEGF)	
Interleukin 1 beta (IL-1β)	Catabolin
Granulocyte colony-stimulating factor (G-CSF)	Colony-stimulating factor 3 (CSF 3)
Epidermal growth factor (EGF)	
Interleukin 10 (IL-10)	Human cytokine synthesis inhibitory factor (CSIF)
Hepatocyte growth factor (HGF)	
Basic fibroblast growth factor (FGF-Basic)	bFGF, FGF2 or FGF-β
Interferon-alpha (IFN-α)	
Interleukin 6 (IL-6)	
Interleukin 12 (IL-12)	
Chemokine (C-C motif) ligand 5 (CCL5)	Regulated upon Activation, Normal T-cell Expressed, and Secreted (RANTES)
Eotaxin (eotaxin-1, eotaxin-2, and eotaxin-3)	Chemokine (C-C motif) ligands 11, 24, and 26 (CCL11, CCL24, and CCL26)
Interleukin 13 (IL-13)	
Interleukin 15 (IL-15)	
Interleukin 17 (IL-17)	Interleukin 17A (IL-17A)
Chemokine (C-C motif) ligand 3 (CCL3)	Macrophage inflammatory protein-1alpha (MIP-1α)
Granulocyte-macrophage colony-stimulating factor (GM-CSF)	
Chemokine (C-C motif) ligand 4 (CCL4)	Macrophage inflammatory protein-1beta (MIP-1β)
Chemokine (C-C motif) ligand 2 (CCL2)	Monocyte chemotactic protein-1 (MCP-1) or small inducible cytokine A2 (SCYA2)
Interleukin 5 (IL-5)	
Interferon-gamma (IFNγ)	
Tumor necrosis factor-alpha (TNFα)	Cachexin or cachectin
Interleukin-1 receptor antagonist (IL-1RA)	
Interleukin 2 (IL-2)	
Interleukin 7 (IL-7)	
Chemokine (C-X-C motif) ligand 10 (CXCL10)	Interferon gamma-induced protein 10 (IP-10) or small inducible cytokine B10 (SCYB10)
Interleukin 2 receptor (IL-2R)	
Chemokine (C-X-C motif) ligand 9 (CXCL9)	Monokine induced by gamma interferon (MIG)
Interleukin 4 (IL-4)	
Interleukin 8 (IL-8)	Neutrophil chemotactic factor

### Statistical methods

All data were analyzed using R 2.15 (http://www.r-project.org) or Graphpad Prism 6.02 (Graphpad Software, Inc., La Jolla, CA). A two-tailed *P* value of <.05 was considered significant for all analyses. Measures of central tendency, variability, and frequency were conducted on demographic variables. Control groups were compared to cases using the unpaired t-test for means/Mann-Whitney test for medians (continuous variables) or the two sample z-test for proportions (categorical variables). Tab-delimited data returned from the Luminex 200 runs were first imported into R. Next, the lower limit of detection for each individual cytokine was set to 1 and the data were log_10_ transformed, due to the inherently non-normal distribution. The corresponding table containing the clinical data was next constructed.

### Ordination

Principal component analysis (PCA) was carried out using the rda() function in R-package *vegan* (Oksanen et al. 2013, *vegan*: Community Ecology Package, http://CRAN.R-project.org/package=vegan). Individual points in PCA plots were connected to their group (case, inpatient control, or outpatient control) centroid using the ordispider() function. A permutational multivariable analysis of variance (MANOVA) was used to test the differences between group centroids (the multi-dimensional mean) using the function adonis().

### Logistic regression

The ordination above helped guide decision-making regarding further analysis. Based on multivariable PCA results, we chose simple logistic regression to identify whether individual inflammatory mediators could predict severe cases vs. non-severe cases and cases vs. outpatient controls. Due to the similar inflammatory profiles in the PCA of cases vs. inpatient controls (discussed further below), we chose to limit clinical variability by using a matched-pair analysis: conditional logistic regression. Inpatient controls were matched to cases by age (± 5 years) and gender. Matching was done using a random number from the sample() function in R when more than one matching possibility existed.

Finally, to incorporate confounding effects an adjusted analysis of inflammatory mediators on the ability to predict CDI cases vs. outpatient controls was performed using multiple logistic regression and included all mediators that were found significant on univariable analysis. This was not done for matched inpatient controls, due to the similarity with CDI cases and non-significant univariable analysis results (discussed further below).

## Results

### Baseline characteristics and initial data analysis

**Figure 2:**
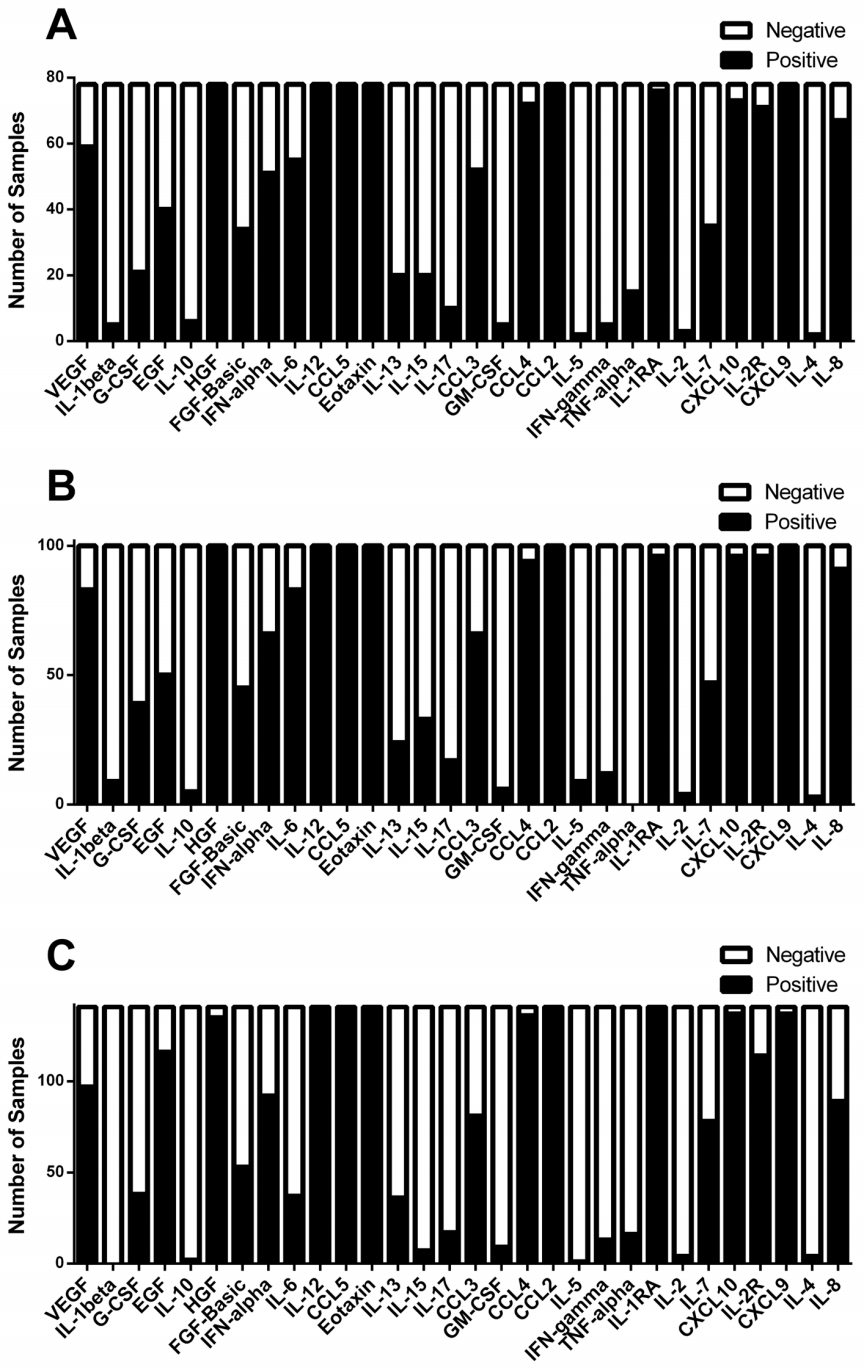
Detectability of circulating inflammatory mediators in *Clostridium difficile* infection (CDI). Results for cases (panel A), inpatient controls (panel B), and outpatient controls (panel C) are shown.

A total of 315 samples were included for analysis, with baseline patient characteristics shown in Table 2. There were 78 cases, 100 inpatient controls, and 137 outpatient controls. All cases tested positive for toxigenic *C. difficile* in stool by our testing algorithm [Figure 1] and were confirmed on culture, save one subject from whom we were unable to culture *C. difficile*. This subject was included in the final analysis as the sample was positive for toxigenic *C. difficile* by PCR for toxin B and the case was clinically compatible with CDI. All control subjects (inpatient and outpatient) had negative culture results for toxigenic *C. difficile*. Outpatient controls were significantly younger than inpatient cases (*P* = .015). Overall and in all three groups, there were more females than males, though the differences between groups did not reach significance (Table 2). There were no significant differences between cases and inpatient controls with regards to Charlson-Deyo score, PPI use, fever, or albumin, though PPI use was present in >70% of subjects in both groups. Cases did have a higher mean white blood cell (WBC) count than controls (*P* = .038). For several of the individual inflammatory mediators (listed in Table 1), many patients had levels below the limits of detection (Figure 2).

**Table 2:** Patient characteristics.

	All	CDI Cases	Inpatient Controls^1^	Outpatient Controls^2^
**Total (%)**	315	78 (24.8)	100 (31.7)	137 (43.5)
**Median Age (range)**	58 (18−88)	59.5 (59−87)	61 (18−85)	50.5 (19−88)
***P*** 3	NA	NA	.772	.015
**Female Gender (%)**	192 (61)	44 (56.4)	59 (59)	89 (65)
***P*** 3	NA	NA	.729	.214
**Median Charlson-Deyo Score (IQR [range])**	1 (0−2.25 [0−7])	1 (1−3 [0−7])	1 (0−2 [0−6])	-
***P*** 3	NA	NA	.038	-
**PPI Use (%)**	131 (73.6)	58 (74.4)	73 (73)	-
***P*** 3	NA	NA	.838	-
**Fever** 4 **(%)**	30 (16.9)	17 (21.8)	13 (13)	-
***P*** 3	NA	NA	.120	-
**Mean WBC** 5 **(SD)**	11.1 (9.8)	12.8 (11.1)	9.8 (8.4)	-
***P*** 3	NA	NA	.038	-
**Mean Albumin (SD)**	3.2 (0.6)	3.2 (0.6)	3.2 (0.6)	-
***P*** 3	NA	NA	.551	-
**Severe CDI** 6 **(%)**	NA	8 (10.3)	NA	NA
**Death** 7 **(%)**	1	1	0	-
**ICU Admission (%)**	15 (4.8)	7 (9)	8 (8)	-
**Colectomy (%)**	2 (0.6)	0	2 (2)	-

*Abbreviations*: CDI, *Clostridium difficile* infection; IQR, interquartile range; NA, not applicable; PPI, proton pump inhibitor; SD, standard deviation; WBC, white blood cell count. Missing data is indicated by a hyphen.

1 Diarrhea, but no CDI.

2 Healthy patients without diarrhea or CDI.

3 *P* values compare control groups to cases and use the unpaired t-test for means/Mann-Whitney test for medians (continuous variables) or the two sample z-test for proportions (categorical variables).

4 Temperature > 38°C.

5 thousands of cells per mm^3^_._

6 Intensive care unit admission, colectomy, or death attributed to CDI within 30 days of diagnosis.

7 All-cause 30-day mortality.

### Ordination of circulating inflammatory mediator expression in C. difficile positive patients vs. inpatient and outpatient controls

**Figure 3:**
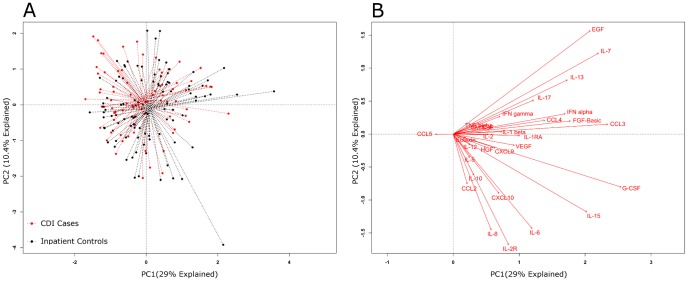
Global systemic inflammatory responses in *C. difficile* infection (CDI) cases and inpatient controls. Principal component analysis (PCA) (panel A) results are shown for CDI cases and inpatient controls. The individual inflammatory mediators’ effects on the PCA were plotted as biplots (panel B). In biplots the arrows indicate the direction of maximum change while the length of arrows represents the magnitude of the change. The PCA centroids were not significantly different by permutational MANOVA testing (*P* = .051).

**Figure 4:**
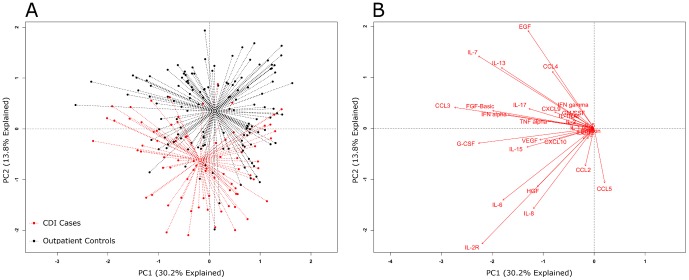
Global systemic inflammatory responses in *C. difficile* infection (CDI) cases and outpatient controls. Principal component analysis (PCA) (panel A) results are shown for CDI cases and outpatient controls. The individual inflammatory mediators’ effects on the PCA were plotted as biplots (panel B). In biplots the arrows indicate the direction of maximum change while the length of arrows represents the magnitude of the change. The PCA centroids were different by permutational MANOVA testing (*P*<.001).

The antibody-linked bead array examining 30 different mediators (Table 1) was used to assay the systemic inflammatory response in plasma samples and this generated a large amount of data, which was first explored by principal component analysis (PCA). Figure 3A depicts a PCA of inflammatory mediator data from cases and inpatient controls; and Figure 4A displays a PCA for cases and outpatient controls. The dotted lines connect each point to its group centroid (the multi-dimensional mean). The position of the centroids indicated that there was an overall difference in the mediators in cases vs. outpatient controls but not vs. inpatient controls.

Next, the differences observed between cases and controls were tested for significance. A permutational MANOVA determined that significant differences existed between cases and outpatient controls (*P*<.001), but not cases and inpatient controls (*P* = .051).

Next, the influences of individual inflammatory mediators on the PCA were determined by analyzing the data in the form of a biplot (Figures 3B and 4B). In PCA biplots, arrows indicate the direction of maximum change while the length of arrows represents the magnitude of the change. Figure 4B indicates that the differences between cases and outpatient controls were driven by higher levels of certain individual mediators: IL-2R, IL-8, IL-6, HGF, CCL2 (MCP-1) and CCL5 (RANTES).

### Results of logistic regression

The above PCA provided evidence that patients with CDI had measurable systemic inflammatory responses compared with outpatient controls, and that these differences could be driven by certain specific inflammatory mediators. To refine our understanding of which specific mediators associated with the presence and severity of CDI, we conducted unadjusted analyses using logistic regression. Only CCL5 associated with CDI cases vs. matched inpatient controls (OR 1.98, 95% CI 1.06 – 3.68, *P* = .031; Table 3). Several cytokines predicted the presence of CDI compared with outpatient controls (Table 3): HGF, IL-2R, IL-8, IL10, IL15, and CCL5. CDI was associated with low levels of EGF, eotaxin, and CCL4 (MIP1β). Eight cases met CDC criteria for severe CDI and the most significant predictor of severe CDI vs. non-severe CDI was an elevated IL-8 level (OR 5.92; 95% CI 1.13 – 31.1, *P* = .036), though eotaxin (OR 0.09, 95% CI 0.01 – 0.97, *P* = .047) and IL-6 (OR 3.12, 95% CI 1.05 – 9.28, *P* = .041) were also significant while the other 27 mediators tested were not (data not shown).

**Table 3:** Simple logistic regression results for serum inflammatory mediators (cytokines, chemokines, and growth factors) in patients with *Clostridium difficile* infection (CDI) vs. matched inpatient controls who tested negative for CDI and asymptomatic outpatient controls (all units in log^­^_10_ pg/mL).

	Matched Inpatient Controls^1^			Outpatient Controls^1^		
Inflammatory Mediator	OR	95% CI	*P*	OR	95% CI	*P*
VEGF	0.58	0.32−1.05	.073	1.27	0.76−2.14	.364
IL-1â	0.85	0.46−1.57	.607	N/A	N/A	>.99
G-CSF	0.81	0.61−1.07	.137	0.98	0.74−1.31	.907
EGF	0.87	0.59−1.28	.484	**0.38**	**0.27**−**0.55**	**<.001**
IL-10	1.13	0.62−2.05	.699	**2.60**	**1.03**−**6.59**	**.044**
HGF	1.20	0.63−2.26	.579	**14.78**	**6.10**−**35.8**	**<.001**
FGF-Basic	0.96	0.61−1.51	.864	1.18	0.79−1.77	.413
IFN-á	0.99	0.66−1.49	.949	0.99	0.67−1.44	.944
IL-6	0.71	0.47−1.08	.111	**3.94**	**2.60**−**5.97**	**<.001**
IL-12	1.16	0.37−3.60	.797	1.97	0.49−7.96	.343
CCL5	**1.98**	**1.06**−**3.68**	**.031**	**2.72**	**1.54**−**4.83**	**.001**
Eotaxin	0.58	0.22−1.53	.273	**0.29**	**0.10**−**0.84**	**.023**
IL-13	1.12	0.73−1.72	.611	1.02	0.73−1.44	.896
IL-15	0.84	0.60−1.17	.299	**2.49**	**1.55**−**4.00**	**<.001**
IL-17	0.87	0.56−1.34	.516	1.10	0.69−1.76	.681
CCL3	1.02	0.69−1.52	.910	1.30	0.93−1.80	.119
GM-CSF	1.77	0.59−5.28	.308	1.18	0.52−2.63	.694
CCL4	0.74	0.44−1.24	.256	**0.45**	**0.27**−**0.76**	**.003**
CCL2	0.71	0.28−1.82	.475	**2.94**	**1.21**−**7.13**	**.017**
IL-5	0.43	0.14−1.32	.138	1.88	0.36−9.82	.456
IFN-ã	0.75	0.39−1.45	.394	0.82	0.43−1.57	.551
TNF-á	N/A	N/A	>.99	1.67	0.85−3.30	.139
IL-1RA	0.85	0.48−1.50	.576	0.52	0.23−1.19	.120
IL-2	0.89	0.39−2.01	.770	1.07	0.48−2.41	.865
IL-7	0.86	0.57−1.30	.464	0.75	0.55−1.03	.076
CXCL10	0.84	0.51−1.40	.511	**2.35**	**1.12**−**4.92**	**.024**
IL-2R	0.66	0.43−1.01	.057	**2.13**	**1.47**−**3.08**	**<.001**
CXCL9	0.74	0.37−1.47	.389	**1.96**	**1.01**−**3.82**	**.047**
IL-4	0.80	0.34−1.84	.592	0.92	0.40−2.14	.848
IL-8	0.67	0.41−1.09	.104	**3.44**	**2.12**−**5.58**	**<.001**

1 Versus patients with CDI.

In adjusted analysis (multiple logistic regression) of inflammatory mediators’ ability to predict cases vs. outpatient controls, several retained significance (Table 4). Once again, CCL5 significantly associated with cases (OR 4.48, 95% CI 1.50−13.4, *P* = .007) as did HGF (OR 6.59, 95% CI 1.89−23.1, *P* = .003). Cases were again associated with low EGF (OR 0.29, 95% CI 0.16−0.54, *P*<.001).

**Table 4:** Multiple logistic regression results for serum inflammatory mediators (cytokines, chemokines, and growth factors) in patients with *Clostridium difficile* infection (CDI) vs. outpatient asymptomatic controls who tested negative for CDI (all units in log^­^_10_ pg/mL).

Inflammatory Mediator	OR	95% CI	*P*
**EGF**	**0.29**	**0.16**−**0.54**	**<.001**
IL-10	1.04	0.23−4.77	.959
**HGF**	**6.59**	**1.89**−**23.1**	**.003**
**IL-6**	**2.14**	**1.14**−**4.02**	**.018**
**CCL5**	**4.48**	**1.50**−**13.4**	**.007**
**Eotaxin**	**0.12**	**0.02**−**0.95**	**.045**
IL-15	1.89	0.91−3.92	.087
CCL4	0.73	0.33−1.59	.427
CCL2	0.15	0.02−1.47	.104
CXCL10	0.82	0.26−2.59	.739
IL-2R	1.25	0.73−2.13	.412
CXCL9	2.24	0.56−9.04	.257
IL-8	1.95	0.96−3.97	.067

## Discussion

The present study newly demonstrates a broad systemic inflammatory response that accompanies CDI in hospitalized patients, a finding that could accelerate the discovery of biomarkers for improving diagnosis, prognosis, or response to treatment. The results of this investigation revealed that the host response to CDI in hospitalized patients was complex, yet similar to that observed in other inpatients with *C. difficile*-negative diarrheal illness.

Comparative analyses of circulating inflammatory mediator profiles were initially performed using PCA. PCA is a method of multivariable analysis that uses a mathematical transformation to reduce a high-dimensional problem (one that has many variables) to a low-dimensional representation of the data. This is then displayed from its most informative viewpoint (where the largest differences can be visualized). PCA showed that the centroids were different between the inflammatory profiles of cases and asymptomatic outpatient controls, but not between cases and inpatient controls (Figures 3A and 4A). Since the PCA biplots (Figures 3B and 4B) showed that certain mediators may be driving these differences, we explored this further through unadjusted and adjusted analysis.

The initial unadjusted analysis of individual inflammatory mediators and the subsequent adjusted analysis showed that a number of individual mediators were different between cases and outpatient controls, but that the most robust predictors were HGF and CCL5, with EGF being inversely associated with cases (Table 4). CCL5 was also the only mediator that distinguished between cases and matched inpatient controls (Table 3), as our analysis otherwise suggested a non-specific systemic inflammatory response common to diarrheal illness, but not specifically to CDI. In our analysis of severe disease, IL-8 had the most robust predictive ability, although both eotaxin and IL-6 were also associated with more severe infection. However, these results were limited by a low number of severe cases (only eight) and the likelihood of a type II error masking any potential association with other mediators. Thus, our analysis draws attention to several inflammatory mediators that can be the target of future study including mediators of epithelial integrity/regrowth (HGF and EGF) and chemotactic factors for leukocytes (CCL5 and IL-8).

The damage to the luminal epithelium of the colon seen in CDI [7] may be driving the elevation in HGF we observed—hepatocyte growth factor is secreted by mesenchymal cells, acts primarily on epithelial cells, and has been shown to have a major role in wound healing and tissue regeneration [23], [24]. The inverse association we observed between elevated levels of EGF and CDI cases may be due to a protective effect of EGF on the epithelial barrier. EGF promotes the integrity and maintenance of the epithelial barrier, and this has led some to study its role in CDI. It has been shown to diminish epithelial cell damage and disruption of cytoskeletal F actin in response to toxins A and B [25] and impair the decline in transepithelial resistance caused by toxin B exposure [26].

The role of CCL5 in the pathogenesis of CDI in humans has been little described and it is intriguing that our analysis suggests a CDI-specific elevation, given the difference observed between cases and inpatient controls. CCL5 is chemotactic for T cells, eosinophils, and basophils, playing an active role in recruiting leukocytes into inflammatory sites [27], [28], [29]. Animal studies suggest that CCL5 is a biological mediator in the pathogenesis of CDI. A mouse study that exposed animals to *C. difficile* toxin A implicated CCL5 (and its receptor, CCR1) as an important mediator of acute intestinal inflammation during infection [30]. Targeting CCL5 signaling with a selective antagonist reduced neutrophilic inflammation in the toxin A-exposed mice [30]. Thus, CCL5 warrants further attention in future human studies of CDI, for its role in disease pathogenesis and as a potential biomarker.

Although the number of patients with severe CDI was limited, we noted a significant association of severe disease with elevations in circulating eotaxin, IL-6, and IL-8 levels compared with non-severe infection. The chemokine IL-8, which is also known as neutrophil chemotactic factor, has been previously shown to be elevated in fecal samples from patients with CDI and the levels associate with disease severity [14], [15], though this effect may be common to other diarrheal illnesses such as inflammatory bowel disorder [31]. IL-8 may play a central role in the pathogenesis of severe CDI, as there exists an IL-8 gene polymorphism that has been associated with increased susceptibility to severe CDI [32]. The present results expand this paradigm by suggesting that serum IL-8 levels may also associate with severe CDI. Should this result be validated in future studies, serum IL-8 could be an easily measureable biomarker that would assist clinicians in risk stratification and aggressive therapeutic interventions. For example, the decision to use vancomycin in lieu of metronidazole, which is recommended by current guidelines [33], or the pursuit of colectomy-sparing loop ileostomy procedures [34], may in part be guided by such a biomarker.

This study was limited by a lack of information regarding the presence of concomitant immunosuppression, infections, or other inflammatory conditions that could affect systemic cytokine levels and we are unable to comment on how this could have influenced our results. Also, some of the demographic variability (Table 2), including a preponderance of female gender in all three groups and the younger age of outpatient controls, could have acted as important confounders and we did not analyze this. Though it is possible that our clinical laboratory’s testing algorithm may have misclassified subjects, a prior analysis of our algorithm has shown the specificity to be 98% and the negative predictive value 99% [35]. We cultured all samples and were unable to confirm only one case, which was positive by PCR for *tcdB* and clinically compatible with CDI. Although the number of subjects included in the study overall was not large, the baseline characteristics (Table 2), including the demographics and the higher mean WBC in patients with CDI, suggest that our patient population is rather typical for a hospitalized cohort with CDI in the United States, and supports the generalizability of our findings. Finally, for certain individual inflammatory mediators there was a low rate of detectability in serum (Figure 2), and it is possible that this influenced our results more than the actual values of the mediators in samples where they were detected.

A common diagnostic challenge is accurately ruling out CDI in patients with diarrhea from other causes who are also colonized with a toxigenic *C. difficile* strain. This is a particular concern in patients diagnosed on the basis of nucleic acid amplification tests for *C. difficile* toxin B or toxin A, without confirmation of the presence of actual toxin in the stool [36]. A detailed understanding of the systemic changes in inflammatory mediators that accompany CDI could reveal infection-specific biosignatures capable of differentiating true infection from colonization in hospitalized patients with diarrhea. This could even include mediators not tested in this study, such as procalcitonin, which has been previously shown to be associated with severe CDI [37]. This is an area for future research and was not examined in the present study.

In summary, this study, measuring the peripheral circulating levels of 30 different inflammatory mediators in CDI, sheds new light on details of the systemic inflammatory response that occurs during infection. The results highlight several specific mediators of interest, which could guide future research. This work further underscores the previously identified link between IL-8 and CDI severity.
